# Supplementary figures and images for: NEDD4 regulates ubiquitination and stability of the cell adhesion molecule IGPR-1 via lysosomal pathway
Source: J Biomed Sci. 2021 May 7;28:35. doi: 10.1186/s12929-021-00731-9 (PMC8103646; doi:10.1186/s12929-021-00731-9)

S. Figure 1

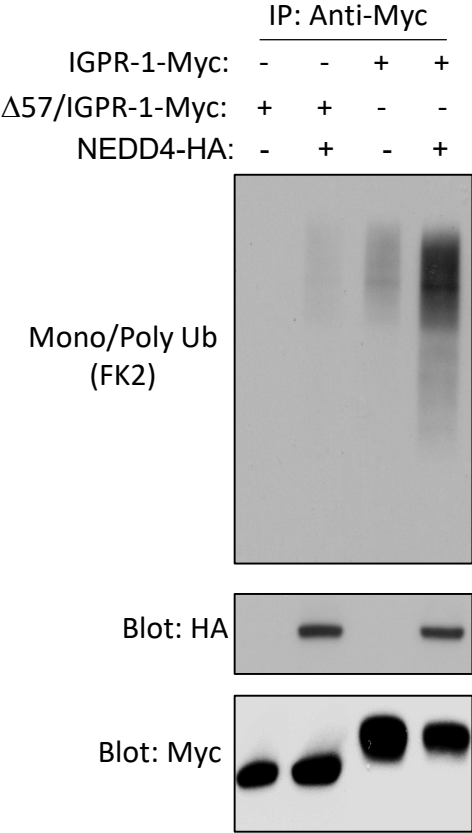

Supplement: Supplementary file 1 — Additional file 1: S. Figure 1. Deletion of proline-rich motifs on IGPR-1 inhibits its NEDD4-mediated ubiquitination. [file 12929_2021_731_MOESM1_ESM.pdf]

S. Figure 2

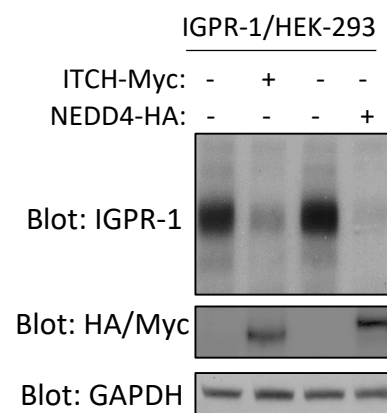

Supplement: Supplementary file 2 — Additional file 2: S. Figure 2. Co-expression of NEDD4 or ITCH with IGPR-1 promotes downregulation of IGPR-1. [file 12929_2021_731_MOESM2_ESM.pdf]

S. Figure 3

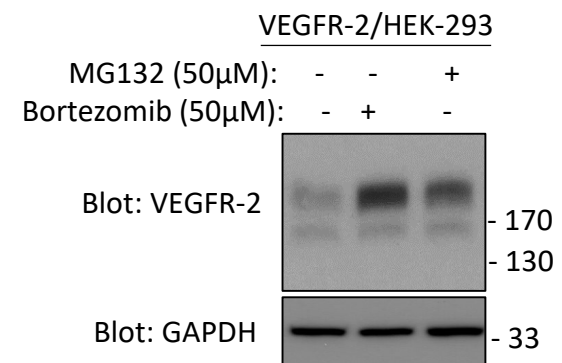

Supplement: Supplementary file 3 — Additional file 3: S. Figure 3. Effect of 26S-proteasome inhibitors on VEGFR-2 downregulation. [file 12929_2021_731_MOESM3_ESM.pdf]

S. Figure 4

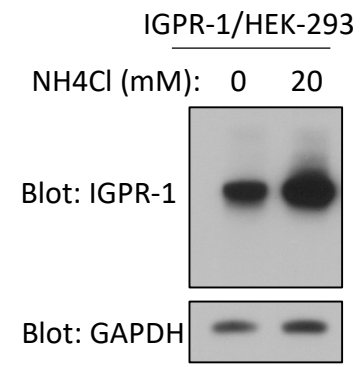

Supplement: Supplementary file 4 — Additional file 4: S. Figure 4. Ammonium chloride (NH4Cl) increases IGPR-1 levels. [file 12929_2021_731_MOESM4_ESM.pdf]

S. Figure 5

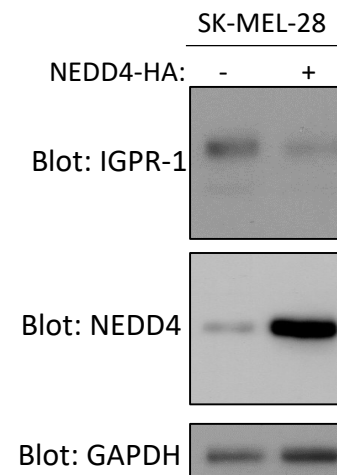

Supplement: Supplementary file 5 — Additional file 5: S. Figure 5. Expression of NEDD4 in SK-MEL-28 cells downregulate IGPR-1 expression. [file 12929_2021_731_MOESM5_ESM.pdf]
